# Sialic Acid Binding Sites in VP2 of Bluetongue Virus and Their Use during Virus Entry

**DOI:** 10.1128/JVI.01677-21

**Published:** 2022-01-12

**Authors:** Weining Wu, Polly Roy

**Affiliations:** a Department of Infection Biology, Faculty of Infectious and Tropical Diseases, London School of Hygiene and Tropical Medicine, London, United Kingdom; Instituto de Biotecnologia/UNAM

**Keywords:** BTV/entry/receptor/sialic acid, bluetongue virus, receptor, sialic acid, virus entry

## Abstract

Bluetongue virus (BTV), a member of the *Orbivirus* genus, is transmitted by biting midges (gnats, Culicoides sp.) and is one of the most widespread animal pathogens, causing serious outbreaks in domestic animals, particularly in sheep, with high economic impact. The non-enveloped BTV particle is a double-capsid structure of seven proteins and a genome of 10 double-stranded RNA segments. Although the outermost spike-like VP2 acts as the attachment protein during BTV entry, no specific host receptor has been identified for BTV. Recent high-resolution cryo-electron (cryoEM) structures and biological data have suggested that VP2 may interact with sialic acids (SAs). To confirm this, we have generated protein-based nanoparticles displaying multivalent VP2 and used them to probe glycan arrays. The data show that VP2 binds α2,3-linked SA with high affinity but also binds α2,6-linked SA. Further, Maackia amurensis lectin II (MAL II) and Sambucus nigra lectin (SNA), which specifically bind α2,3-linked and α2,6-linked SAs, respectively, inhibited BTV infection and virus growth in susceptible sheep cells while SNA alone inhibited virus growth in Culicoides-derived cells. A combination of hydrogen deuterium exchange mass spectrometry and site-directed mutagenesis allowed the identification of the specific SA binding residues of VP2. This study provides direct evidence that sialic acids act as key receptor for BTV and that the outer capsid protein VP2 specifically binds SA during BTV entry in both mammalian and insect cells.

**IMPORTANCE** To date no receptor has been assigned for non-enveloped bluetongue virus. To determine if the outermost spike-like VP2 protein is responsible for host cell attachment via interaction with sialic acids, we first generated a protein-based VP2-nanoparticle, for the multivalent presentation of recombinant VP2 protein. Using nanoparticles displaying VP2 to probe a glycan array, we identified that VP2 binds both α2,3-linked and α2,6-linked sialic acids. Lectin inhibitors targeting both linkages of sialic acids showed strong inhibition to BTV infection and progeny virus production in mammalian cells; however the inhibition was only seen with the lectin targeting α2,6-linked sialic acid in insect vector cells. In addition, we identified the VP2 sialic acid binding sites in the exposed tip domain. Our data provides direct evidence that sialic acids act as key receptors for BTV attachment and entry in to both mammalian and insect cells.

## INTRODUCTION

Nonenveloped orbiviruses with complex capsid structures represent an intriguing system for understanding virus entry mechanisms. Although the *Orbivirus* genus belongs to the *Reoviridae* family, these viruses are uniquely vectored to wild and domestic animal species (e.g., sheep, cattle, horses, deer, etc.) by arthropods (gnats, ticks, or mosquitoes). Bluetongue virus (BTV), the prototype of the genus, with 28 serotypes, is one of the most widespread animal pathogens and acts as an important representative of this class of large non-enveloped viral pathogens. BTV is transmitted by *Culicoides* species, gnats (biting midges), between its animal hosts, and often causes serious periodic outbreaks particularly in sheep and cattle, with high economic impact.

BTV is structurally highly complex with a genome of 10 double-stranded RNA (dsRNA) segments (S1–S10) enclosed by four layers of different proteins. Ten genomic segments encode seven structural proteins (VP1–VP7) and four non-structural proteins (NS1–NS4). The outer capsid is formed by two consecutive layers of proteins, the outermost VP2 and the slightly less exposed VP5, and both proteins attach to an underlying VP7 layer which coats the VP3 core. The remaining three structural proteins (VP1, VP4, and VP6) form the enzymatic interior of the virus together with genomic RNA. Recent atomic-resolution structures have revealed that 120 VP2 molecules form 60 trimers and that each monomer consists of three distinct domains (hub, body, and tip), displayed as triskelion-like spikes, each blade of which (*viz.*, the tip domain) protrudes 4 nm from the surface of the particles while the hub and body base sits on the underlying VP5 trimers (1). The structural configuration of VP2 is consistent with it biological functions, as it is the host attachment protein, and also the viral antigenic determinant of serotype specificity (2). Upon attachment, VP2 facilities the clathrin-mediated endocytosis of virion particles. In the early endosome, VP2 senses the low pH via a unique zinc finger and consequently changes conformation and dissociates from the virion particle. The remnant particle, with VP5 still attached, then proceeds to the late endosome where the acidic pH triggers drastic conformation change to enable membrane penetration and release of the viral core into the cytoplasm (1, 3, 4).

While much molecular and structural study has been undertaken on the action of VP2 and VP5 in cell entry, to date no receptor has been assigned for BTV or any other orbiviruses, although our previous studies have suggested that cell surface sialic acids, and possibly additional receptors may be involved (1, 5). Arboviruses generally do not rely on a single specific host receptor—rather they use common cellular ligands, such as Ca^2+^-dependent (C-type) lectins, immunoglobulin fragment crystallisable-gamma (Fcγ) receptors, and tyrosine-protein kinase receptor Axl (6).

Sialic acids are known to be involved for attachment and entry of other non-enveloped and enveloped viruses, such as adenoviruses, rotaviruses, and influenza viruses (7–9) and our previous studies indicated that BTV binds glycophorin A, which contains high levels of sialic acids (10). Further, the lectin wheat germ agglutinin (WGA), which binds specifically to N-Acetyl-d-glycosamine (GlcNAc), had been shown to block BTV binding to the cells (5, 10). However, there is no direct evidence that VP2 binds sialic acids and if so what types of sialic acid may serve as BTV ligands during virus entry.

In this report, we seek to determine that sialic acid is a cellular ligand for BTV and that VP2 is directly responsible for this interaction. To do this, we first generated recombinant VP2 as protein nanoparticles (Fc-VP2-PNP) to increase avidity through multivalent interactions as reported for coronavirus spike protein (11). Using these nanoparticles, it was possible to obtain direct evidence of specific interaction between VP2 and specific sialic acids, particularly its specificity for α2,3 and α2,6 linkages. Further, we determined the sialic acid preference used by BTV for mammalian versus vector Culicoides cells. Lastly, we identified the sialic acid binding sites and the key residues of VP2 responsible for virus attachment to the host cells.

## RESULTS

### Multivalent presentation of recombinant VP2 protein on the nanoparticles.

**FIG 1:**
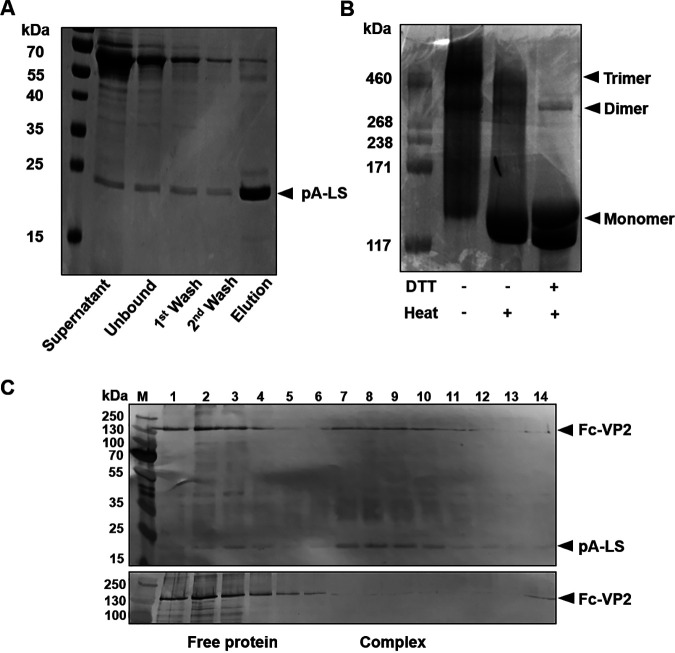
Fc-VP2 and the pA-LS nanoparticle form a complex. (A) SDS-PAGE with Coomassie blue staining confirmed the purity and correct size of purified recombinant pA-LS protein. (B) SDS-PAGE with Coomassie blue staining showing the native Fc-VP2 protein largely in trimeric form in the absence of heat and DTT reducing agent. (C) Migration of Fc-VP2 in the presence or absence of pA-LS nanoparticles in a 14%–45% continuous glycerol gradient showing the formation of VP2 and nanoparticle complex Fc-VP2-PNP in fractions 7 to 10 of a total of 14 fractions collected from top to bottom of the gradient.

BTV is capable of agglutination of a variety of animal and human red blood cells (RBCs) *in vitro*. The hemagglutination (HA) specificity of certain erythrocytes has been reported for different BTV serotypes with variable intensity, indicating the activity is serotype dependent (12, 13). Our previous use of recombinant purified BTV-10 VP2 confirmed that VP2 is responsible for binding to glycophorin on RBCs and hemagglutanation activity (10). However, the hemagglutination activity of purified VP2, as a soluble protein, is generally very low when compared with the virion particle (10) and is too weak to investigate the interaction of VP2 with sialic acid receptors (14). To overcome the low affinity of VP2 binding to sialic acids, we designed multicopy VP2 self-assembled into a nanoparticle similar to that reported for MERS-CoV S1 protein, which then showed enhanced hemagglutination activity (11). The nanoparticle is a 60-meric self-assembled particle of lumazine synthase (LS) from the hyperthermophile Aquifex aeolicus. The N-terminus of LS is fused with domain B of protein A (pA) from Staphylococcus aureus which has a high affinity for the Fc fragment of human IgG (15). Accordingly, a construct was made to express the pA-LS nanoparticle protein as a secreted protein from transfected HEK293T cells with an added N-terminal streptavidin tag to facilitate affinity purification. The purified nanoparticles were analyzed by SDS-PAGE followed by Coomassie blue staining. The protein content of the nanoparticles showed a monomer molecular mass of ∼25 kDa, as predicted (Fig. 1A). To allow binding of VP2 to the nanoparticle, the amino terminus of VP2 was tagged with the Fc sequence from human IgG and a streptavidin tag II (WSHPQFEK) for affinity purification and the subsequent fusion protein expressed using the baculovirus expression system. Analysis of purified Fc-VP2 protein by SDS-PAGE showed that VP2 largely existed in its trimeric form, however, in the presence of reducing agent or heat treatment the protein converted to the monomeric form (Fig. 1B). To prepare VP2-nanoparticles, 6 μg of pA-LS nanoparticles and 20 μg of Fc-VP2 were incubated for 30 min at room temperature and the formation of VP2 and nanoparticle complex was analyzed by glycerol gradient ultracentrifugation. A clear shift was apparent of Fc-VP2 from the top layer to the middle-lower layer of the gradient in the presence of pA-LS compared with Fc-VP2 in the absence of pA-LS, indicating the formation of a nanoparticle complex (Fig. 1C). We calculated the amounts of pA-LS and Fc-VP2 in fractions 7 to 10 by densitometry, suggesting a molar ratio between 1:0.2 to 1:0.29 of pA-LS and Fc-VP2 in the complex.

### VP2-nanoparticles (Fc-VP2-PNPs) enhance the hemagglutination and the glycophorin binding activities.

**FIG 2:**
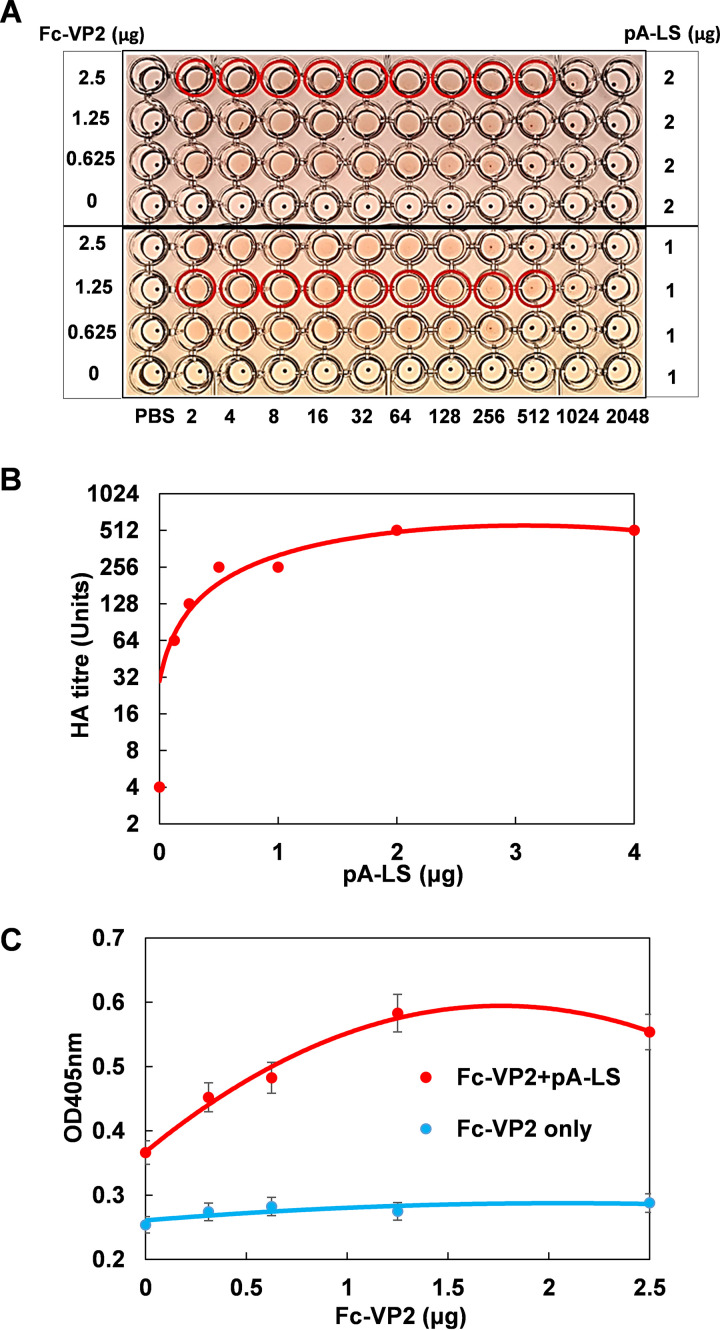
Optimization of binding between Fc-VP2 and pA-LS nanoparticle. (A) The HA titers of variable amounts of Fc-VP2 combined with 1 μg or 2 μg of pA-LS were measured using 0.25% of washed sheep RBCs. Highlighting shows the maximum HA titers with the combination of 2.5 μg of Fc-VP2 and 2 μg of pA-LS or 1.25 μg of Fc-VP2 and 1 μg of pA-LS. (B) The HA titers of 2.5 μg of Fc-VP2 combined with variable amounts of pA-LS were measured using 0.25% of washed sheep RBCs. Highlighting shows the maximum HA titer obtained with the combination of 2.5 μg of VP2-Fc and 2 μg of pA-LS. (C) Glycohrorin A (1 μg) binding of variable amounts of Fc-VP2 plus 2 μg of pA-LS or Fc-VP2 alone were measured by ELISA showing the significantly higher binding affinity by the complex compared with Fc-VP2.

To examine if the VP2 nanoparticle could hemagglutinate sheep RBCs, a combination of different amount of Fc-VP2 and 1 or 2 μg of pA-LS was optimized for hemagglutination activity. The combination of 1 μg pA-LS and 1.25 μg Fc-VP2 (equivalent to a molar ratio of 1:0.25) exhibited the maximum hemagglutination (HA) titer of 512 with sheep RBCs. In contrast, nanoparticles lacking Fc-VP2, as a control, failed to show any hemagglutination activity (Fig. 2A). When 2.5 μg of Fc-VP2 was incubated with decreasing amounts of nanoparticles (1, 0.5, 0.25, and 0.125 μg) the HA titers also decreased. However, increasing the Fc-VP2 amount in the nanoparticles to 4 μg did not significantly increase the HA titer, indicating that nanoparticles have been saturated for their maximum binding capacity (Fig. 2B). The optimized combination of Fc-VP2 and pA-LS, which exhibited the maximum HA titer, suggested that approximately five of Fc-VP2 trimers can be accommodated on each of the 60-mer pA-LS nanoparticles forming Fc-VP2-PNP. Fc-VP2 alone without nanoparticles exhibited a very low HA titer, of only ∼4 units (Fig. 2B) consistent with a previous study (10) and an inability to cross-link the RBCs. Thus, nanoparticles presenting VP2 have significantly increased hemagglutination activity.

To determine whether the multivalent Fc-VP2 nanoparticles could increase the interaction of VP2 to human glycophorin A, the predominant sialoglycoprotein on human RBCs, we used an ELISA. A glycophorin A-coated 96-well ELISA plate was incubated with mixtures of 2 μg pA-LS and different amounts of purified Fc-VP2 or Fc-VP2 alone as before. While binding of VP2 alone was minimal, binding of the Fc-VP2-PNPs was significantly high and was dose-dependent, with the maximum binding at the molar ratio about 1:0.2 of pA-LS and Fc-VP2 (Fig. 2C). These data confirm that VP2-nanoparticles significantly increase sialic acid binding capacity when compared to VP2 protein alone.

### Does VP2 recognize specific sialic acid for cell attachment?

**FIG 3:**
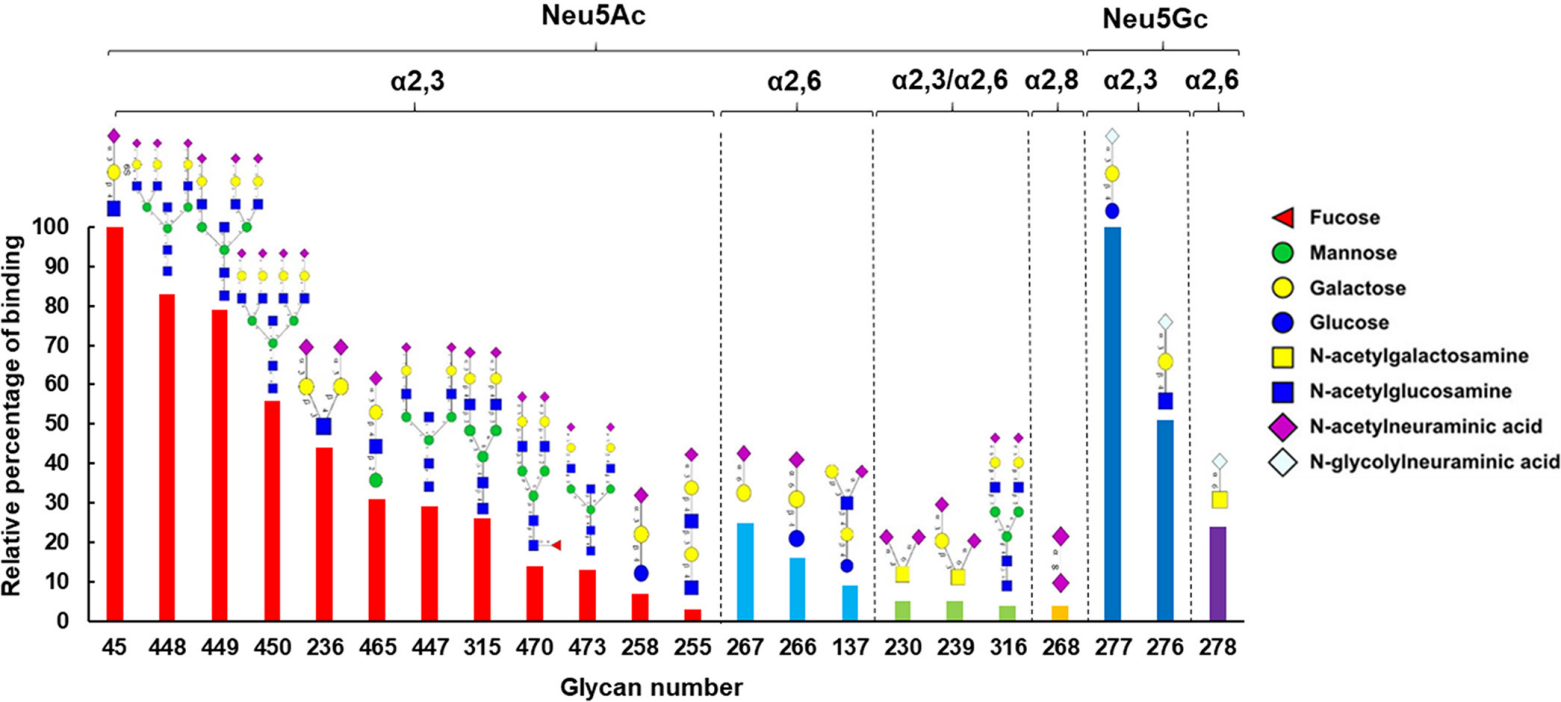
VP2 nanoparticles, assembled as described, were used for analysis of glycan binding to glycan arrays of the Functional Glycomics Gateway as described. Relative binding to a subset of glycans, of 562 total, is shown. Symbols to the right of the figure identify individual sugars present within the glycan chains shown.

The enhanced sialic acid binding affinity of Fc-VP2-PNPs led us to use these particles to identify the VP2 preference for specific sialic acid linkages. To this end, we probed an array of 562 glycans (16) with the Fc-VP2-PNPs and compared the binding profile with uncoated PNPs alone as control. As shown in the Fig. 3, the results showed clearly that there is a preferential binding to sialylated glycans by the Fc-VP2-PNPs complex compared with the PNPs alone, which did not bind to any sialylated glycan structures (Table S1), indicating the specific interaction between VP2 and sialylated glycans.

Based on the glycan array data, VP2 bound to both Neu5Ac and Neu5Gc sialoglycans with a preference for Neu5Ac (Fig. 3). Further, it preferentially bound to the short, unbranched α2,3-linked sialotrisaccharides (glycan #45 and #277) and α2,6-linked (glycan #267, #266, and #278) sialoside, as well as to long, branched α2,3-linked sialosides with a minimum extension of an N-Acetyllactosamine (LacNAc) tandem repeats. A low level of binding was observed to a number of α2,3/α2,6-linked and α2,8-linked sialosides but the preferential binding was clearly to α2,3-linked sialosides (Fig. 3). Our data are consistent with earlier reports that both α2,3- and α2,6-linked sialic acids could be utilized as receptors by BTV for attachment during its entry into the host cells.

### BTV infection is inhibited by α2,3- and α2,6-linked sialic acid binding lectin competitors.

**FIG 4:**
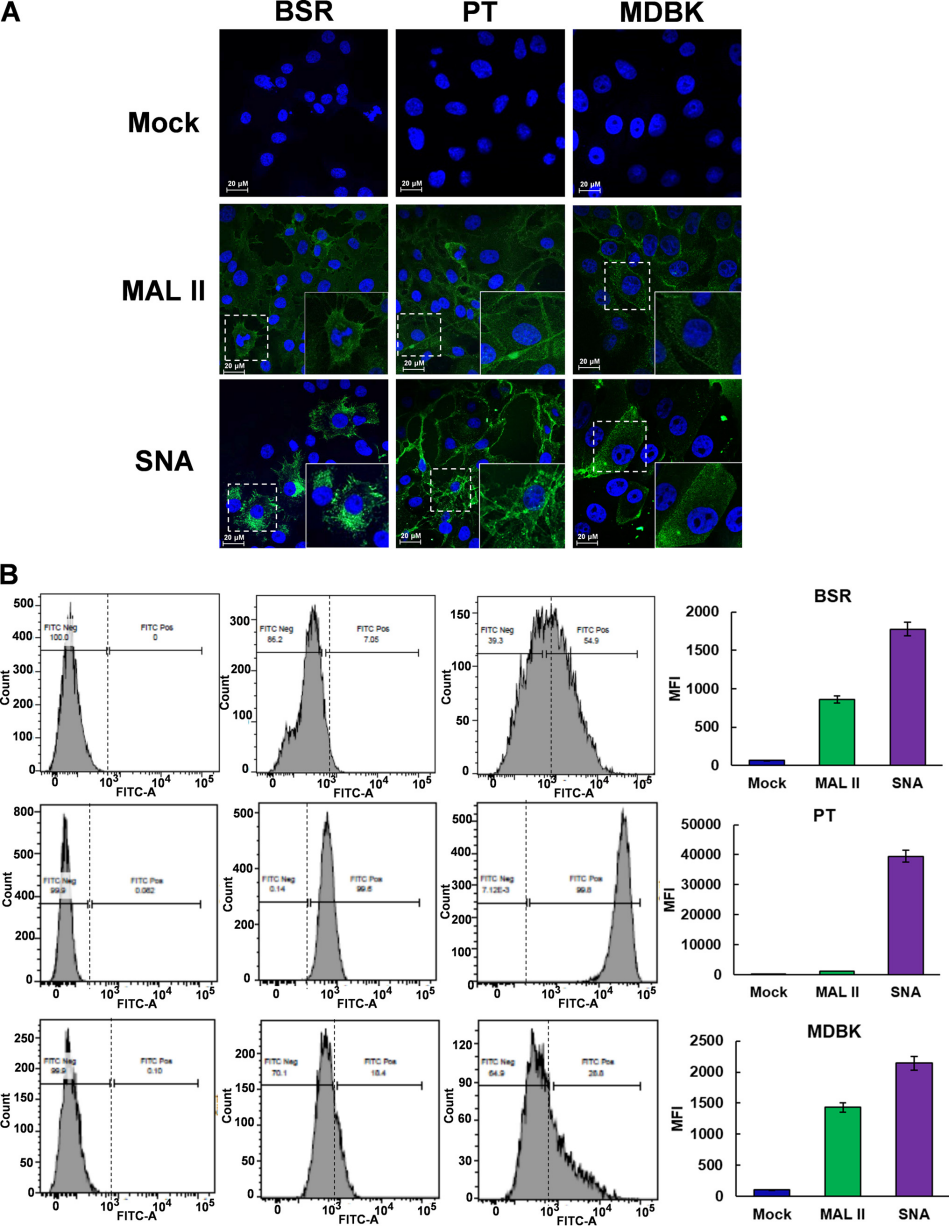
Distribution of α2,3- and α2,6-linked sialic acids on the surface of BSR, PT, and MDBK cells. (A) Cell surface sialic acids visualized by confocal immunofluorescence microscope using FITC-conjugated MALII and SNA, which specifically bind to α2,3- and α2,6-linked sialic acids, respectively. Sialic acids is shown in green and nuclei stained with Hoechst shown in blue. The insets at the bottom right corner show an enlarged version of single cell staining from the area enclosed by the dashed line. Scale bar = 20μM. (B) FITC-conjugated MALII and SNA labeled cell surface sialic acids measured by flow cytometry shown in histogram (left) and then quantified and shown as median fluorescence intensity (right).

To investigate further if BTV used different sialic acids for different host cells, a series of mammalian cells were examined for BTV infection in the presence of specific lectin inhibitors, Maackia amurensis lectin II (MALII), which binds α2,3-sialic acid and SNA, which prefers to bind α2,6-sialic acid. Prior to an assessment of SA specificity, we first examined the distribution of α2,3- and α2,6-linked sialic acids on the surface of three different mammalian cells, BSR (BHK-21 derived) cells that are used for BTV infection routinely in the laboratory and the two different BTV-susceptible host cell lines, PT (sheep) cells and MDBK (steer) cells by immunofluorescence microscopy using FITC-conjugated MALII and SNA. All three types of cells showed a predominant distribution of α2,6-linked sialic acids over α2,3-linked sialic acids, although PT cells exhibited abundant distribution of both α2,3- and α2,6-linked sialic acids compared with the other two mammalian cell lines (Fig. 4A). The data was further confirmed by flow cytometry analyses. As shown in Fig. 4B, α2,6-linked sialic acids of all three cell lines exhibited higher level of median fluorescence intensity (MFI) of α2,6-linked sialic acids compared to that of α2,3-linked sialic acids.

**FIG 5:**
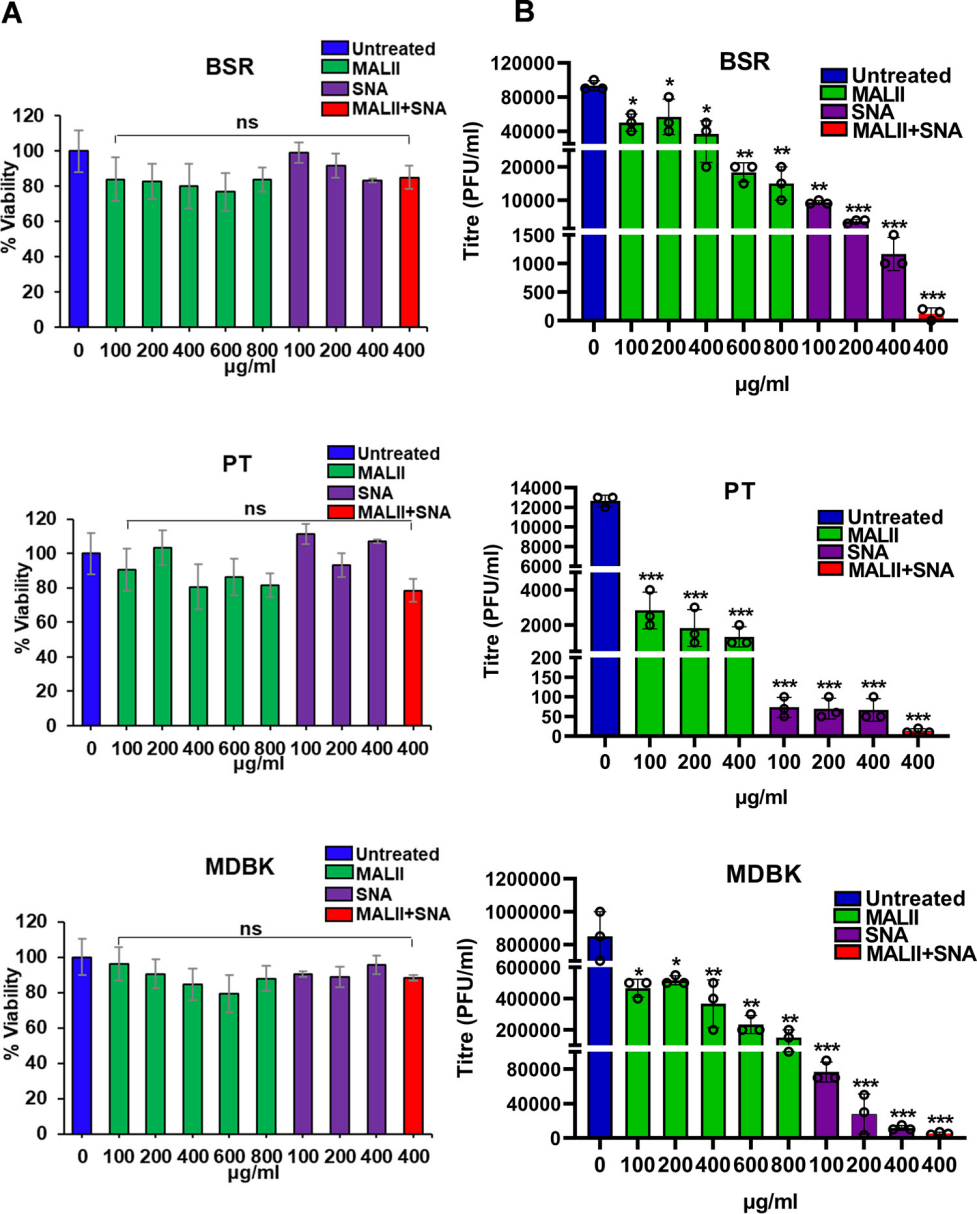
Inhibitory effect of specific lectins on BSR, PT, and MDBK cells. (A) MTT cytotoxicity assay showed no significant cytotoxicity effect at the concentrations of lectins tested. (B) Inhibitory effect of MALII or SNA or a combination of both at different concentration on progeny BTV production in virus infected BSR, PT, and MDBK cells. Statistical analysis: two-way ANOVA test (*n* = 3) *P < 0.05, **P < 0.01, ***P < 0.001, ns *P > *0.05.

**FIG 6:**
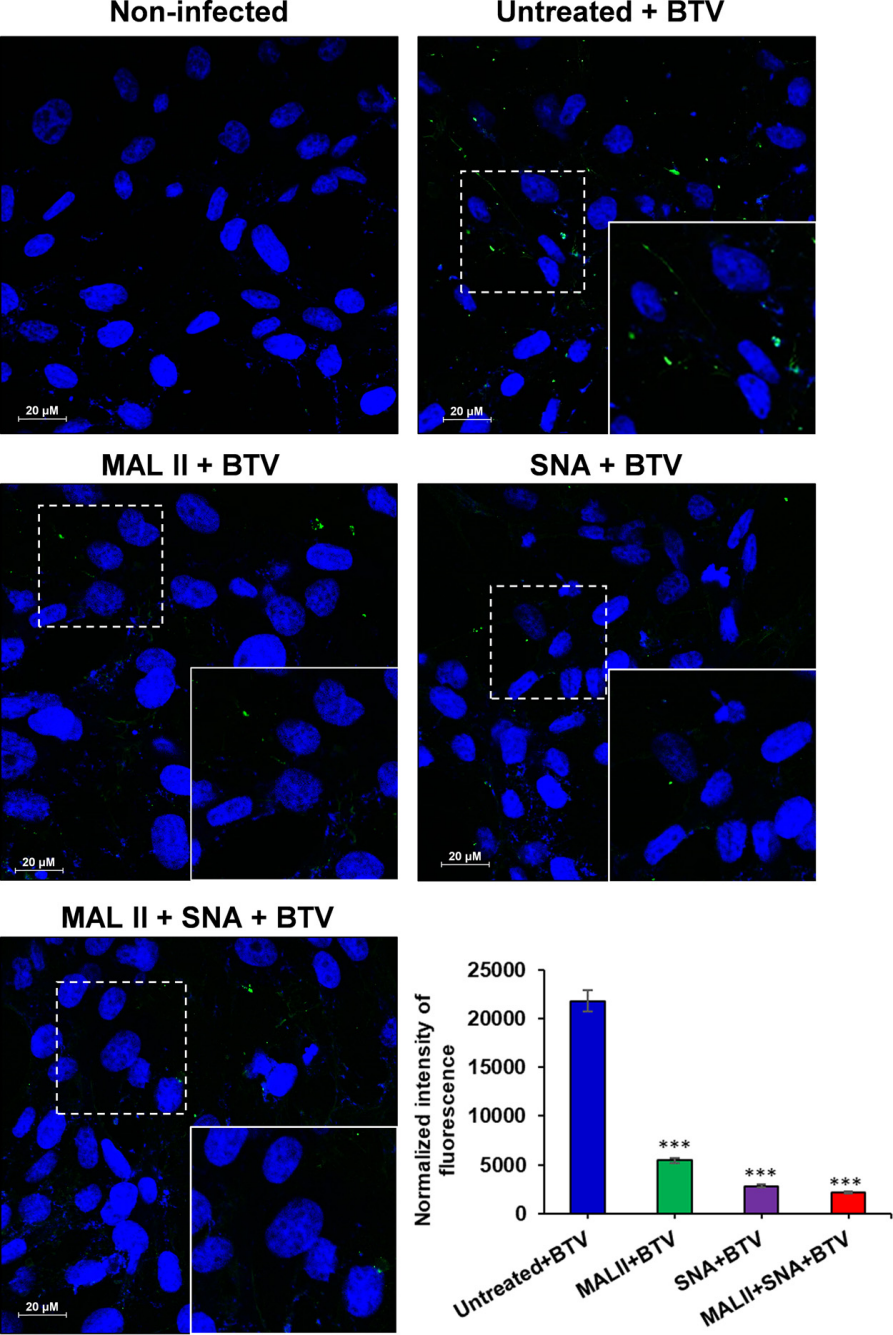
Viral binding to PT cells was inhibited in the presence of 400 μg/mL of MALII or 400 μg/mL of SNA or both lectins at 400 μg/mL of each lectin. Lectin treated cells were incubated with BTV at an MOI of 10 at 4°C. The bound viruses were labeled with anti-BTV antibody and visualized by confocal immunofluorescence microscope. BTV is shown in green and nuclei stained with Hoechst shown in blue. The insets at the bottom right corner show an enlarged version of cells staining from the area enclosed by the dashed line. The normalized fluorescence intensity of cell surface BTV was measured by Fiji software and plotted as histogram. Two-way ANOVA test ***P < 0.001.

All three cells were then preincubated with 100, 200, 400, 600, or 800 μg/mL of MALII or SNA, and a combination of both lectins at 400 μg/mL of each lectin, prior to infection with BTV. An MTT (2,3-bis-(2-methoxy-4-nitro-5-sulfophenyl)-2H-tetrazolium-5-carboxanilide) cytotoxicity assay showed that there was no significant cytotoxicity at the concentrations tested (Fig. 5A). Both MALII and SNA lectin competitors inhibited progeny virus production in a dose-dependent manner in all three types of cells. At 400 μg/mL concentration, MALII showed approximately 63%, 88%, and 59% inhibition of virus infection in BSR, PT, and MDBK cells, respectively, while SNA showed more than 90% inhibition in all cells. With the combination of both lectins at the concentration of 400 μg/mL, inhibition was almost 100% in all three mammalian cell lines (Fig. 5B). These results are consistent with the observed distribution of α2,3- and α2,6-linked sialic acids in the three cell lines, and suggest that BTV could take advantages of both type of sialic acids for entering mammalian host cells. To prove that blocking cell surface sialic acids prevents viral binding, we compared the viral binding with PT cells in the presence or absence of lectins. PT cells were pretreated with 400 μg/mL of MALII, 400 μg/mL of SNA, or both lectins at 400 μg/mL of each lectin, and viral binding was performed at 4°C and bound viruses were visualized by confocal immunofluorescence microscope. The level of virus on the cell surface was also quantified by measuring the fluorescence intensity. Lectin treatment resulted in a significant decrease of viral binding to PT cells (Fig. 6). This further confirmed that BTV binds cell surface sialic acids during entry.

**FIG 7:**
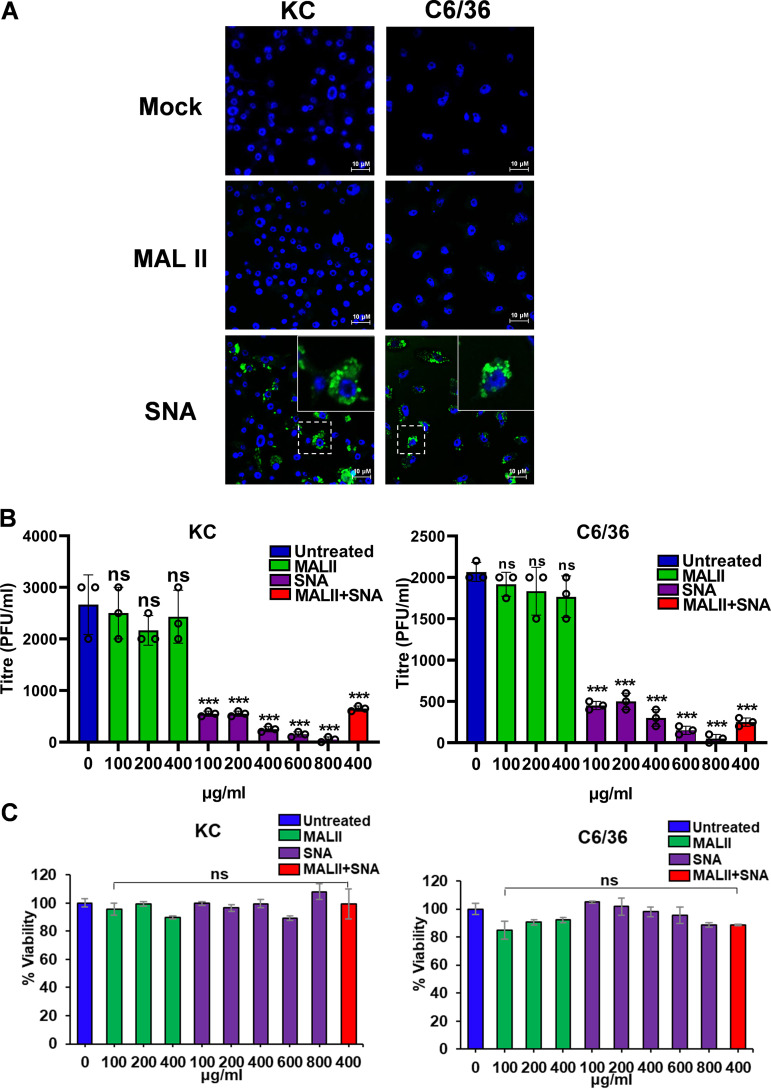
Inhibitory effect of specific lectins on KC and C6/36 cells. (A) Immunofluorescence showing the distribution of α2,3- and α2,6-linked sialic acids on the surface of KC and C6/36 cells using FITC-conjugated MALII and SNA which specifically bind to α2,3- and α2,6-linked sialic acids, respectively. Sialic acids shown in green and nuclei stained with Hoechst shown in blue. The insets at the top right corner show an enlarged version of single cell staining from the area enclosed by the dashed line. Scale bar = 10μM. (B) Inhibitory effect of MALII and SNA or a combination at different concentration on progeny virus production in BTV infected KC and C6/36 cells. (C) MTT cytotoxicity assay showed no significant cytotoxicity effect at the concentrations of lectins tested. Statistical analysis: two-way ANOVA test (*n* = 3) ***P < 0.001, ns *P > *0.05.

To address if BTV entry into insect cells also utilizes sialic acids, we similarly examined the distribution of α2,3- and α2,6-linked sialic acids on the surface of Culicoides-derived cells (KC). In addition, because mosquito C6/36 cells are also susceptible to BTV infection in the laboratory (17), we included the C6/36 cells in the analysis. Both KC and C6/36 cells appeared to express exclusively α2,6-linked sialic acids, as demonstrated by immunofluorescence staining with the specific lectins (Fig. 7A). Further, BTV infection of these cells in the presence of the two inhibitors confirmed further that MALII, the inhibitor of α2,3-linked sialic acids had almost no inhibitory effect, while SNA, the inhibitor of α2,6-linked sialic acids, inhibited 90% of progeny virus production in a dose-dependent manner (Fig. 7B). To eliminate the possibility that the effect was due to cell cytotoxicity, we performed the MTT assay, which showed no impact on the viability of cells (Fig. 7C). These data suggest that BTV utilizes α2,6-linked sialic acid for entry into the KC and C6/36 cells.

Taken together, the data demonstrate that α2,3- and α2,6-linked sialylated glycans play a major receptor role for BTV entry in both mammalian and insect cells. However, the role of other receptors cannot be excluded, particularly for KC and C6/36 cells, as previous studies have indicated that VP2 is not essential for infection and that the integrin binding RGD motif present on the VP7 protein could be responsible for the process (18, 19).

### Putative sialic acid binding sites of VP2 revealed by mass spectrometry analysis.

**FIG 8:**
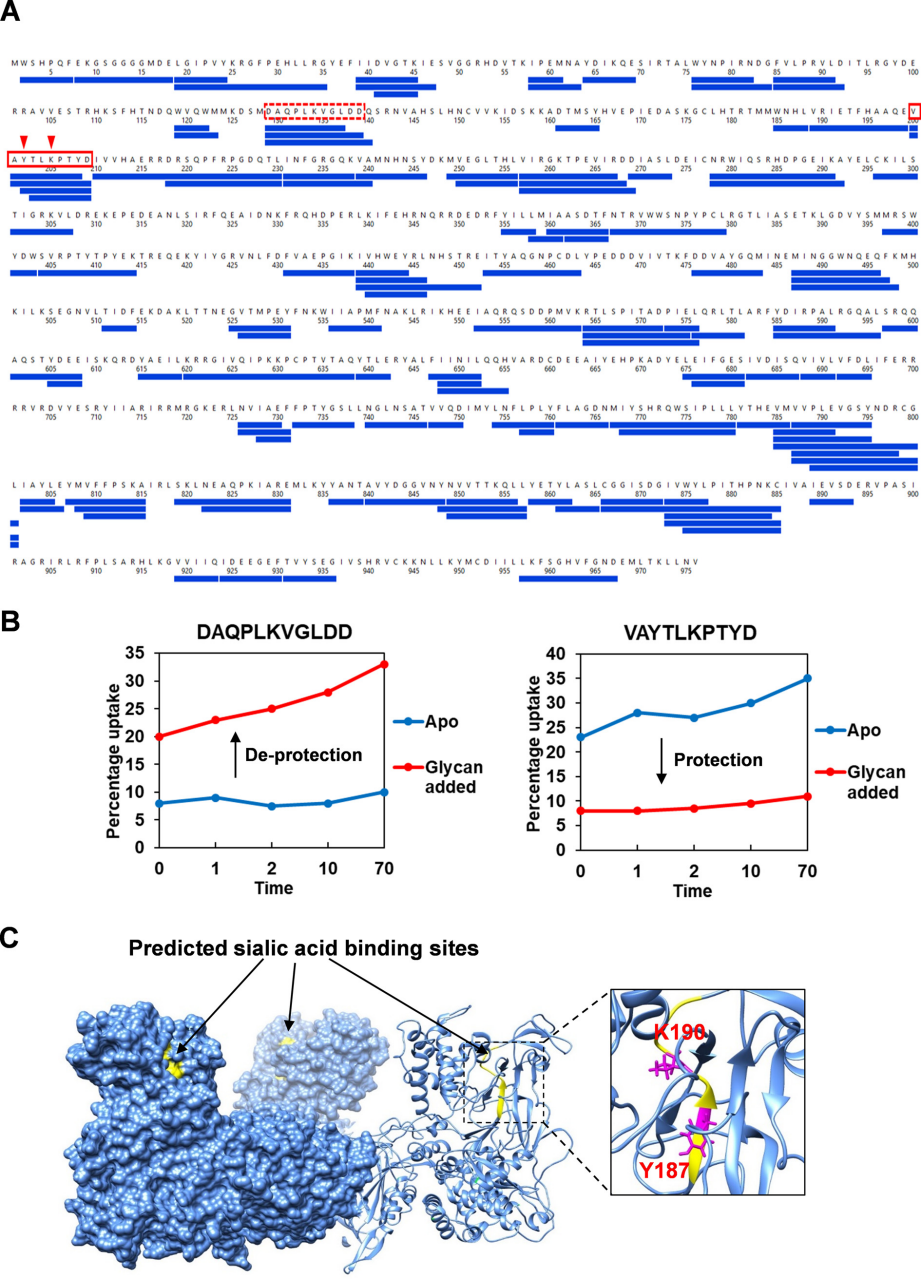
HDX-MS reveals potential sialic acid binding site in VP2. (A) Coverage of peptides from VP2 identified (blue boxes) by HDX-MS after digestion with pepsin and aspergillopepsin in series. (B) Peptide DAQPLKVGLDD and peptide VAYTLKPTYD showing protection (solid line red box) and deprotection (hatched red box), respectively, in the presence of the sialoglycan 3′-sialyl-N-acetyllactosamine in HDX. Conserved amino acid residues Y187 and K190 within peptide VAYTLKPTYD are predicted to bind sialic acid (red arrow). (C) Predicted sialic acid binding sites (yellow) and residues Y187 and K190 (pink) are located at the tip domains of VP2 trimer.

**FIG 9:**
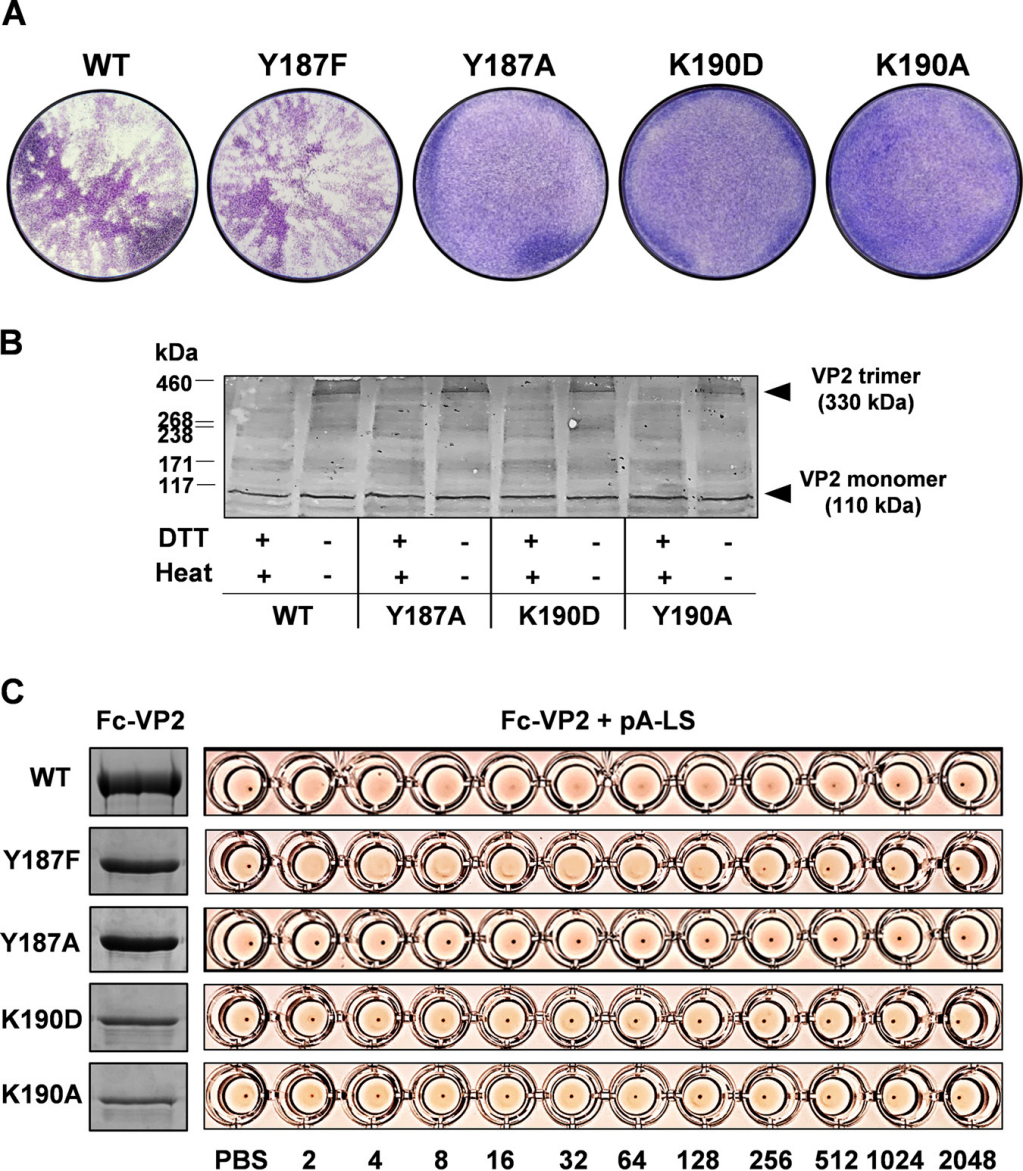
Mutations at two highly conserved residues result in failure of virus recovery and loss of VP2 hemagglutination activity. (A) BTV reverse genetics containing each mutant S2 or WT S2 were performed in BSR cells. Plaque assay shows the virus recovery failed with three mutants—Y187A, K190D, and K190A—while Y187F developed virus plaques with a similar phenotype to that of WT at 72 h posttransfection. (B) SDS-PAGE followed by Western blotting with a rabbit anti-VP2 antibody showing similar expression level and trimerization of VP2 mutant (Y187A, K190D, and Y190A) proteins with that of WT VP2 protein in BSR cells transfected with capped WT or mutant S2 RNA segments. (C) SDS-PAGE with Coomassie blue staining confirmed the purity and correct size of purified recombinant Fc-VP2 mutant (Y187F, Y187A, K190D, and K190A) protein similar to the WT Fc-VP2 protein (left). However, recombinant Fc-VP2 mutant (Y187A, K190D, and K190A) protein incorporated into polyvalent nanoparticles was unable to agglutinate sheep RBCs in contrast to the WT and Y187F mutant Fc-VP2 protein (right).

In order to locate the sialic acid binding sites on VP2, hydrogen deuterium exchange mass spectrometry (HDX-MS) was used. Deuterium exchange with hydrogen atoms in VP2 protein prepared in D_2_O buffer, followed by quenching, proteolysis, and peptide detection by MS allows an assessment of the dynamic change of VP2 protein conformation upon SA binding. Purified VP2 was incubated with 3′-sialyl-N-acetyllactosamine (Neu5Acα2,3Galβ1-4GlcNAc) and digested by pepsin and aspergillopepsin prior to HDX-MS analysis. After carefully optimizing the quenching condition, a total number of 126 peptides were identified, yielding an overall VP2 sequence coverage of 63.1% (Fig. 8A). Ligand-induced alteration of the H/D exchange rate was only observed in two peptides, peptide 114–124 (DAQPLKVGLDD) of VP2 was deprotected, while peptide 185–194 (VAYTLKPTYD) was protected in the presence of the glycan, suggesting that peptide VAYTLKPTYD is involved in sialic acid binding (Fig. 8B). To confirm the data, we introduced substitution mutations in this peptide, targeting specifically the two highly conserved residues Y187 and K190 followed by attempted virus rescue by reverse genetics. Of two substitutions at Y187, one to phenylalanine and the other to alanine, only the Y187F mutant was recovered as viable virus. The substitution of Y187A failed to recover the virus. Similarly, mutations at K190 to aspartic acid or to alanine failed to recover infectious progeny virus consistent with Y187 and K190 being critical for virus fitness (Fig. 9A) and also consistent with a role in receptor binding. To verify that these lethal mutations did not alter the overall structure and key function of VP2, mutant proteins Y187A, K190D, and K190A were expressed in BSR cells by transfecting with capped mutant S2 RNA segments. The expression of three mutant proteins and ability to trimerize, was not compromised, indicating that these mutations did not perturb the overall structure of the protein (Fig. 9B). To substantiate this hypothesis, each of these mutant proteins was expressed as recombinant baculovirus and each purified protein was incorporated into polyvalent nanoparticles. However, none of these three mutant protein nanoparticles showed any hemagglutination activity with sheep RBCs, in contrast to that of the WT or Y187F mutant proteins (Fig. 9C). These results are consistent with a role for the targeted residues in both SA binding and infection.

## DISCUSSION

BTV generally has very low receptor binding avidity, which is reflected by its low hemagglutinin activity (10). Thus, the identification of BTV receptor binding using the virus itself or the purified recombinant VP2 protein has been quite challenging. We have circumvented that by successfully generating protein nanoparticle displaying with multivalent presentation of VP2. Such VP2-PNP had significantly enhanced avidity of VP2 interaction with glycans. This is the first tailored design of protein nanoparticle scaffolds for multivalent presentation of a non-enveloped viral glycoprotein, which has been proven a powerful tool for studying of virus-receptor interaction.

We used glycan array to identify that recombinant VP2 binds both α2,3- and α2,6-linked sialic acids with an overall higher affinity to α2,3-linked sialic acids. During a natural BTV transmission cycle, VP2 mediates BTV binding to sheep erythrocytes via blood meals by the Culicoides vector. Sheep erythrocytes show almost exclusively α2,3-linked sialic acid (20). Treatment with MALII resulted in hemagglutination of sheep RBCs but treatment of SNA did not (data not shown). Therefore, the particularly high abundance of α2,3-linked sialic acid on sheep erythrocytes could serve as the primary target of infection.

Glycan array showed that VP2 binds both Neu5Ac and Neu5Gc sialoglycans with α2,3- and α2,6-linkage. Trypsin treatment had shown no effect on the ability of BTV hemagglutination of human erythrocytes (21) and the ability of VP2 binding to L929 cells (10), suggesting that VP2 binds to O-linked glycans containing sialic acids. Despite an overall stronger binding to α2,3-linked sialic acid, inhibition of α2,6-linked sialic acid demonstrated a significant effect on progeny virus production in three different mammalian cells corresponding to a predominant distribution of α2,6-linked sialic acid on all three mammalian cell lines. Baby hamster kidney cells (BHK-21) generally express high levels of Neu5Ac and low levels of Neu5Gc, while ovine and bovine cells express high levels of Neu5Gc but less Neu5Ac (22). All three cell lines could be effectively infected by BTV, though VP2 showed a preference of binding to Neu5Ac sialoglycan. This evidence demonstrates that both linked Neu5Ac and Neu5Gc sialic acids allow efficient virus attachment and entry into host mammalian cells. Moreover, treatment of MALII or SNA or a combination of both lectins effectively prevented viral binding to the surface of PT cells, confirming that both α2,3- and α2,6-linked sialic acids are directly involved in virus attachment and entry. Notably, PT cells is more sensitive to the treatment with both lectin competitors targeting α2,3- and α2,6-linked sialic acids compared with BSR and MDBK cells. The data was consistent with the immunofluorescence confocal microscopy analysis, which confirmed that there is greater amount of both linked sialic acids on the surface of PT cells; therefore, they are more susceptible to BTV infection. This may also explain why BTV has a wide cell tropism in tissue culture cells. However, the very specific host species tropisms of BTV indicated that there are likely some other factors involved.

Insect cells generally do not produce sialylated glycoproteins (23). However, the presence of a functional CMP-Sia synthase (CSAS) had been detected in Aedes Aegypti, and dengue virus (DENV) was found to recognize α2,6-linked sialic acid structures on the surface of mosquito tissues, suggesting its potential key roles during the early DENV-vector interactions (24). Because the complete genomic sequence of Culicoides sonorensis is now available (25), we were able to identify two transcripts CSON006402-1 and CSON006950-1 encoding two 426aa and 427aa undefined proteins, which are predicted to function as *beta*-galactoside *alpha*-2,6-sialytransferase by PANTHER algorithm (http://www.pantherdb.org/). In this study, we demonstrated that both Culicoides KC and Aedes Albopictus C6/36 cells express exclusively α2,6-linked sialic acid, which plays a major role for BTV entry although other putative receptors may be involved.

Our previous structural data predicted the existence of putative sialic acid binding sites on the hub domain of VP2 and other putative binding sites on the tip domain for unknown receptors (5). Unlike other dsRNA viruses, such as rotavirus or mammalian reovirus (26), the BTV VP2 trimer has a triskelion shape composed of three tip domains protruding from a central hub domain, which is essential to prevent activation of the underlying membrane penetration protein VP5. Here we identified the peptide VAYTLKPTYD at the interface of body and tip domains protected in the presence of sialoglycan Neu5Acα2,3Galβ1-4GlcNAc in the HDX-MS. Therefore, we predicted that the sialic acid binding site is most likely located at the peptide VAYTLKPTYD and may extend into the connecting region in each of the three tip domains (Fig. 8C). We confirmed the conserved residues Y187 and K190 are critical for sialic acid binding using virus recovery assay in combination with mutagenesis and hemagglutination assay.

This study revealed sialic acids as a functional receptor and the different role of α2,3- and α2,6-linked sialic acid for BTV infection in both mammalian cells and insect cells, and also provided biochemical evidence not only to support the structural data but also to reveal dynamic changes of VP2 during receptor binding. Inhibitors targeting specific sialic acid or putative binding sites could be developed as potent antivirals. However, it does not exclude the possibility that other more generic receptors such as heparan sulfate proteoglycan, integrin may also be used by BTV. Whether it is cell or strain dependent remains to be further investigation.

## MATERIALS AND METHODS

### Cloning and protein expression.

Synthetic strep-pA-LS and strep-Fc sequences were purchased from Eurofins Genomics. Strep-pA-LS was ligated into the pCAGG-PM1 vector using the PacI/AflII sites. Strep-Fc and BTV1 VP2 sequences (Protein Data Bank accession number: 3J9D) were inserted into the BamHI site of baculovirus transfer vector pAcYM1 by Gibson Assembly (NEB). HEK293 cells were transfected with pCAG-strep-pA-LS using polyethylenimine (PEI). Five days posttransfection, supernatant was collected and the pA-LS protein was purified using the Strep-Tactin Superflow Plus (Qiagen). Recombinant baculovirus expressing Fc-VP2 was generated by cotransfecting the pAcYM1-strep-Fc-VP2 and Bacmid DNA into Spodoptera frugiperda (*Sf*9) cells. *Sf*9 cells were then infected with recombinant baculoviruses at an MOI of 2 for 48 h at 28°C. Cells was then pelleted, lysed in lysis buffer (50 mM Tris-HCl pH 8.0, 200 mM NaCl, 1 mM EDTA, 1% NP-40), and Fc-VP2 protein was purified from the lysate using the Strep-Tactin Superflow Plus (Qiagen).

### Glycerol gradient ultracentrifugation.

A sample of 200 μl of Fc-VP2 or Fc-VP2-PNP complex containing 10% glycerol were centrifuged on a 15% to 45% continuous glycerol gradient at 55,000 rpm for 1 h at 4°C. Gradient was then fractionated by collecting the samples from top. Each fraction was then analyzed by SDS-PAGE followed by Western blotting using a rabbit anti-strep II tag antibody (Abcam).

### Hemagglutination assay.

Purified Fc-VP2 at various concentrations was incubated with 1 μg or 2 μg of pA-LS for 30 min at room temperature to allow binding of Fc-VP2 to the pA-LS nanoparticles. Next, 25 μl of the nanoparticle complex and 2-fold serial dilutions thereof were mixed with 25 μl of 0.25% washed sheep erythrocytes (Thermo Fisher Oxoid Ltd.) in V-shaped bottom 96-well plates and incubated for 1 h at room temperature as previously described (4). Hemagglutination of RBC was visualized as a lack of sedimentation to a distinct red pellet and HA titers were calculated as the reciprocal of the lowest positive dilution. PBS dilution buffer was used as negative control.

### ELISA based glycophorin binding assay.

Ninety-six-well Nunc MaxiSorp ELISA plates (Thermofisher) were coated with 1 μg of glycophorin A (Sigma) per well diluted in 50 μl of carbonate coating buffer (15 mM Na_2_CO_3_, 36 mM NaHCO_3_, pH 9.6) and incubated overnight at 4°C. The plates were washed with PBS with 0.05% Tween 20 (PBST) and blocked with 5% skimmed milk in PBST for 1 h at room temperature. Different amounts of Fc-VP2 alone or Fc-VP2-PNPs complex formed with 2 μg of pA-LS were then added to allow binding to glycophorin A. Bound VP2 was detected with a rabbit anti-VP2 pAb and then alkaline phonsphatase-labeled anti-rabbit secondary antibody (Sigma). The reaction was developed with the substrate *p*-nitrophenyl phosphate disodium (Thermo Scientific), and the optical density was determined at 405 nm.

### Glycan array analysis.

A sample of 2 μg of pA-LS and 2 μg of Fc-VP2 or 2 μl of pA-LS alone as negative control were mixed in 50 μl of PBS with 0.1% BSA and incubated for 30 min at room temperature to allow formation of Fc-VP2-PNPs. Additional PBS supplemented with Tween 20 was added to make the final volume of 70 μl containing 0.05% Tween 20 and 0.1% BSA. The reaction mixture was applied on the slide (NCFG Glycan Array v3.0) and incubated for 1 h at room temperature in a dark humidified chamber. Slide was washed with PBST, and then incubated with rabbit anti-VP2 antibody diluted at 1:500 in PBST and 0.1% BSA at room temperature for 1 h. After washing, Alexa 647 conjugated goat anti-rabbit IgG (Jackson Immuno Research) at 5 μg/mL in PBST and 0.1% BSA was added and incubated for 1 h at room temperature. Slides were scanned with InnoScan 1100AL scanner (resolution: 5 μm/pixel, Alexa 647: PMT85/Laser Power High) with data processing using Mapix 8.2.7 software (Innopsys, Chicago, IL, USA). For each set of six replicate spots, the mean and SD were calculated after the highest and lowest values were excluded.

### Immunofluorescence.

Monolayer cells grown overnight on the coverslips were fixed in 4% paraformaldehyde and then incubated with FITC-conjugated lectin MAL II or SNA diluted at 1:500 or 1:1000 in PBST for 1 h at room temperature. Nuclei were stained with Hoechst. Images were then captured using the LSM800 inverted confocal microscope (Carl Zeiss Ltd.).

### Flow cytometry analysis.

Cells were trypsinized, resuspended at 1 × 10^6^ in PBS, and fixed with 4% paraformaldyhyde. The fixed cells were incubated with FITC-conjugated lectin MALII or SNA diluted at 1:500 or 1:1,000 in PBS for 1h at room temperature. Measurement of cells without labeling was included as a negative control. Fluorescence for cells was excited with a 488 nm laser on a BD LSR II flow cytometer (BD Biosciences). At least 1 × 10^4^ cells were analyzed for each sample.

### MTT cytotoxicity assay.

Monolayer cells seeded in 96-well plates were treated with 100, 200, 400, 600, or 800 μg/mL of MALII and SNA lectins or a combination of both at 400 μg/mL of each lectin for 2 h prior to addition of 20 μl of 5 mg/mL 2,3-bis-(2-methoxy-4-nitro-5-sulfophenyl)-2H-tetrazolium-5-carboxanilide (MTT, Sigma) to each well. After an incubation for 3 h at 37°C, media was removed and 150 μl of MTT solvent (4 mM HCl, 0.1% NP-40 in isopropanol) was added. Plates were covered, agitated gently on a shaker for 15 min, and then read at 590 nm by a plate reader. The experiment was performed in triplicate and the percentage of cell viability was calculated.

### Lectin competition assay.

Monolayer cells seeded in 48-well plates were treated with 100, 200, or 400 μg/mL of MALII and SNA lectins or a combination of both at 400 μg/mL of each for 1 h at 4°C prior to infection of BTV serotype 1 at an MOI of 1.0 for 24 h at 37°C (mammalian cells) or 28°C (insect cells). Supernatant virus was then collected and virus titer was determined by plaque assay.

### Viral binding assay.

Monolayer cells grown on coverslips were mock-treated or pretreated with 400 μg/mL of MALII, 400 μg/mL of SNA, or both lectins at 400 μg/mL of each lectin for 1 h at 4°C. Viral binding at an MOI of 10 was performed on ice in the absence or presence of lectin inhibitors. After washing off the unbound viruses, bound viruses were labeled with rabbit anti-BTV primary antibody (1:1,000 dilution) and secondary goat anti-rabbit Alexa Fluor 488 (1:2,000 dilution, Thermofisher) and visualized by LSM800 inverted confocal microscope (Carl Zeiss Ltd). A quantitative assessment of the levels of BTV on the cell surface was measured as normalized integrated fluorescence intensity using the Fiji software.

### Hydrogen deuterium exchange mass spectrometry (HDX-MS).

HDX-MS was carried out using an automated HDX robot (LEAP Technologies, Fort Lauderdale, FL, USA) coupled with an M-Class Acquity LC and HDX manager (Waters Ltd., Wilmslow, Manchester, UK). A 30-μl protein solution containing 0.5 μM VP2 and 150 μM 3′-sialyl-N-acetyllactosamine (Neu5Acα2,3Galβ1-4GlcNAc) in equilibration buffer (10 mM potassium phosphate buffer pH 7.4) was added to 135 μl of deuterated buffer (10 mM potassium phosphate buffer pD7.4) and incubated at 4°C for 60, 120, 600, or 4,200 s. Following the labeling reaction, samples were quenched by adding 50 μl of the labeled solution to 100 μl quench buffer (100 mM potassium phosphate, pH 2.4 with formic acid, 0.2% n-Dodecyl-β-d-Maltopyranoside [DDM]). A 50-μl quenched sample was passed through an online proteolysis column containing a 1:1 mixture of immobilized pepsin and aspergillopepsin (Type XIII protease) (NovaBioAssays, USA) at 500 μl/min and a VanGuard Pre-column Acquity UPLC BEH C18 (Waters Ltd.) for 3 min. The resulting peptic peptides were transferred to a C18 column (Waters Ltd.) and separated by gradient elution of 0%–40% MeCN (0.1% vol/vol formic acid) in H_2_O (0.3% vol/vol formic acid) over 7 min at 40 μl/min. The HDX system was interfaced to a Synapt G2Si mass spectrometer (Waters Ltd.). HDMS^E^ and dynamic range extension modes (data independent analysis [DIA] coupled with IMS separation) were used to separate peptide prior to CID fragmentation in the transfer cell. HDX data were analyzed using PLGS (v 3.0.2) and DynamX (v 3.0.0) software supplied with the mass spectrometer. Restrictions for identified peptides in DynamX were as follows: minimum intensity 1,000, minimum products per amino acid 0.3, max sequence length 25, max ppm error 5, file threshold 4/5.
